# The *LNK* Gene Family: At the Crossroad between Light Signaling and the Circadian Clock

**DOI:** 10.3390/genes10010002

**Published:** 2018-12-20

**Authors:** María José de Leone, Carlos Esteban Hernando, Andrés Romanowski, Mariano García-Hourquet, Daniel Careno, Joaquín Casal, Matías Rugnone, Santiago Mora-García, Marcelo Javier Yanovsky

**Affiliations:** 1 Leloir Institute, Biochemical Research Institute of Buenos Aires (IIBBA)– National Scientific and Technical Research Council (CONICET), Av. Patricias Argentinas 435, Ciudad de Buenos Aires C1405BWE, Argentina; mjdeleone@leloir.org.ar (M.J.d.L.); chernando@leloir.org.ar (C.E.H.); georomano@gmail.com (A.R.); mhourquet@leloir.org.ar (M.G.-H.); dcareno@leloir.org.ar (D.C.); jcasal@agro.uba.ar (J.C.); smora@leloir.org.ar (S.M.-G.); 2 The Keck School of Medicine, University of Southern California, Los Angeles, CA 90089, USA; mrugnone@gmail.com

**Keywords:** circadian clock, light signaling, circadian activators, NIGHT LIGHT–INDUCIBLE AND CLOCK-REGULATED, phototropism

## Abstract

Light signaling pathways interact with the circadian clock to help organisms synchronize physiological and developmental processes to periodic environmental cycles. The plant photoreceptors responsible for clock resetting have been characterized, but signaling components that link the photoreceptors to the clock remain to be identified. Members of the family of NIGHT LIGHT–INDUCIBLE AND CLOCK-REGULATED (*LNK*) genes play key roles linking light regulation of gene expression to the control of daily and seasonal rhythms in *Arabidopsis thaliana*. Particularly, *LNK1* and *LNK2* were shown to control circadian rhythms, photomorphogenic responses, and photoperiod-dependent flowering time. Here we analyze the role of the four members of the LNK family in *Arabidopsis* in these processes. We found that depletion of the closely related *LNK3* and *LNK4* in a *lnk1;lnk2* mutant background affects circadian rhythms, but not other clock-regulated processes such as flowering time and seedling photomorphogenesis. Nevertheless, plants defective in all *LNK* genes (*lnkQ* quadruple mutants) display developmental alterations that lead to increased rosette size, biomass, and enhanced phototropic responses. Our work indicates that members of the LNK family have both distinctive and partially overlapping functions, and are an essential link to orchestrate light-regulated developmental processes.

## 1. Introduction

The circadian clock is an endogenous timekeeper that enables plants to synchronize biological processes with daily and seasonal environmental conditions, allowing the allocation of resources during the most beneficial times of the day and the year. This anticipation of daily and seasonal environmental cycles allows plants to optimize internal processes in relation to external conditions, thus providing a fitness advantage [1,2,3,4,5,6].

The circadian clock regulates more than 30% of the transcriptome in *Arabidopsis*, rice, maize, papaya, poplar, and soybean [3,5,7,8,9,10,11,12,13,14]. Circadian-clock regulated genes are central to many relevant physiological processes such as flowering time, light signaling, growth control, metabolic activities, abiotic stress responses, and plant-pathogen interactions [8,15,16,17,18,19,20,21,22,23].

In *Arabidopsis thaliana*, the core of this oscillator is composed of a group of genes that act at different times of the day to regulate the activity and gene expression of other members of this circadian network. In a simplified model, two partially redundant MYB transcription factors, CIRCADIAN ASSOCIATED 1 (*CCA1*) and LATE ELONGATED HYPOCOTYL (*LHY*), show peak expression levels in the morning. These proteins directly repress the expression of all the members of the PSEUDO-RESPONSE REGULATOR (*PRRs*) gene family, which in turn act as repressors of *CCA1* and *LHY* and show sequential peaks in their expression starting with *PRR9* in the morning and ending with *PRR1*, also known as TIMING OF CAB2 EXPRESSION 1 (*TOC1*), in the evening. In addition, the expression of another group of core clock genes, EARLY FLOWERING 3 (*ELF3*), *ELF4*, and LUX ARRHYTMO (*LUX*), peaks in the evening, and their corresponding proteins act together in the so-called Evening Complex (EC), repressing the expression of morning genes such as *PRR9*. This scenario, where the central oscillator is composed of interconnected negative transcriptional and translational feedback loops (TTFLs), is known as the repressilator, a name that highlights the dearth of activators in the circadian network [24,25].

Our understanding of the plant core clock network has improved significantly since the finding that REVEILLE 8 (RVE8), REVEILLE 6 (RVE6), and REVEILLE 4 (RVE4), three members of the CCA1/LHY/RVE family of single MYB transcription factors, function as activators of several evening clock genes, such as *PRR5*, *TOC1*, and *ELF4*. Interestingly, other members of this family, RVE1, RVE2, and RVE7, affect clock outputs, but not the clock itself [26,27,28]. More recently, in an effort to find components linking light signaling pathways and the circadian clock, two novel transcriptional regulators were discovered: NIGHT LIGHT-INDUCIBLE AND CLOCK-REGULATED GENE 1 (LNK1) and LNK2. Although both genes were proposed to act as transcriptional activators, they do not exhibit any known DNA binding domain [29]. Instead, LNK1 and LNK2 interact with the MYB transcription factors CCA1, LHY, RVE4, and RVE8 [30,31]. These activators (RVE4, RVE6, and RVE8) and co-activators (LNK1 and LNK2) show peak expression at mid-morning and promote the expression of *PRR5* and *TOC1* in the afternoon, whose activity contributes not only to complete the downregulation of CCA1/LHY expression at midnight, but also to repress the expression of all the *LNK* genes, starting the cycle again [29,30,32,33].

It is well known that MYB transcription factors regulate gene expression by directly binding to a 9 bp region present in the promoters of target genes, which is known as the Evening Element (EE) [7,34]. The RVE family is not an exception: it has been reported that RVE8, RVE4, RVE6, RVE3, and RVE5 all bind to the EE motif, suggesting a certain degree of functional redundancy among the members of the RVE family [32,35]. This hypothesis was further corroborated by the observation that, in contrast to the mild 1-h period lengthening phenotype observed in single *rve8* mutants, triple *rve4;rve6;rve8* mutants display a circadian period 4 h longer than the wild-type (WT) [33]. More recently, the degree of functional redundancy of the remaining members of the RVE8 clade, RVE3 and RVE5, was assessed. The *rve3;rve4;rve5;rve6;rve8* mutants display only minor alterations in circadian rhythms in comparison with *rve4;rve6;rve8* mutants; however, these quintuple mutants exhibit severe alterations in the modulation of light inputs to the clock and in the control of plant growth at several developmental stages. Whereas the RVE8 clade represses hypocotyl elongation in seedlings in a day-length dependent manner, in the adult vegetative stage, the *rve3;rve4;rve5;rve6;rve8* mutant presents larger cells and increased leaf area, as well as higher total plant biomass compared not only to WT plants, but also to triple *rve4;rve6;rve8* mutants [36].

While these results bring out the relevance of the functional redundancy of the RVE8 clade, no study of this nature has been conducted yet for the LNK family of co-activators. This group includes LNK1 and LNK2 along with their closest homologs, LNK3 and LNK4. LNK1 and LNK2 are proteins of about 66 kDa with 35% sequence identity. LNK3 and LNK4 are smaller (≈30 kDa each), with 60% sequence identity and with one third of conserved positions also shared with LNK1 and LNK2. Homologs for LNK1, LNK2, and LNK3/LNK4 can be found throughout land plants, including nonvascular plants. LNK1 and LNK2 play an essential role in the proper function of the circadian clock, but the loss of either LNK3 or LNK4 function alone did not confer any obvious clock defect [29,30].

Here we show that, unlike the RVE8 clade, the *lnk1;lnk2;lnk3;lnk4* quadruple mutants (*lnkQ*) display a 1.3-h and 4-h period lengthening in comparison to *lnk1;lnk2* double mutants and WT plants, respectively, while *lnk3;lnk4* double mutants *(lnk3;4)* do not display circadian alterations at all. In addition, *lnk* quadruple mutants display altered leaf shape, bigger rosettes, and significantly enhanced biomass accumulation compared to the *lnk1;lnk2* double mutants (*lnk1;2*), resembling what is seen with the *rve* multiple mutants [36]. We also found a strong hyponastic phenotype in *lnk1;lnk2;lnk3;lnk4* quadruple mutants, which is not present in *lnk1;lnk2* double mutants and is likely due to alterations in phototropic responses. Taken together, our findings provide another example of alterations in circadian clock components that result in enhanced plant growth and biomass accumulation, independently of their effect on flowering time, unveiling new paths for crop improvement.

## 2. Materials and Methods

### 2.1. Plant Material

All the mutants used in this study were in the Columbia (Col-0) accession background. The double mutant *lnk1-1*;*lnk2-1* (*LNK1*/AT5G64170 and *LNK2*/AT3G54500) has been described in Rugnone et al. [29]. The *lnk3-1* and *lnk4-1* mutants (*LNK3*/AT3G12320 and *LNK4*/AT5G06980) were identical to those used in Xie et al. [30]. The double mutant *lnk3;lnk4* was obtained by crossing single mutants and the quadruple *lnkQ* (*lnk1-1*;*lnk2-1;lnk3-1;lnk4-1*) mutant was generated by introgressing the *lnk3-1* and *lnk4-1* alleles into the *lnk1;lnk2* mutant background. Verification of combinatorial mutants was performed via PCR analysis. Primers used for genotyping were previously reported [29,30].

### 2.2. Growth Conditions

Plants were grown on soil at 22 °C under long days (LD; 16-h light/8-h dark cycles; 80 μmol·m^−2^ s^−1^ of white light), 12:12 days (LD 12:12; 12-h light/12-h dark cycles; 80 μmol·m^−2^ s^−1^ of white light), short days (SD; 8-h light/16-h dark cycles; 140 μmol·m^−2^ s^−1^ of white light) or continuous light (LL; 50 μmol·m^−2^ s^−1^ of white light), depending on the experiment.

### 2.3. Circadian Leaf Movement Analysis

For leaf movement analysis, plants were grown under 16-h light/8-h dark cycles until the appearance of the first pair of leaves. This period is referred to as the entrainment period. In order to measure circadian rhythms in leaf movement, plants were transferred to continuous white light (20 μmol·m^−2^ s^−1^) at 22 °C. The position of the first pair of leaves was recorded every 2 h for 5–6 days using digital cameras and the leaf angle was determined using ImageJ software [37]. Period estimates were calculated with Brass 3.0 software (Biological Rhythms Analysis Software System, available from http://www.amillar.org) and analyzed with Fast Fourier Transform Non-linear Least Squares (FFT-NLLS) using Brass 3.0 software. These experiments were performed in triplicate, with n = 8 for each genotype. The statistical analysis was done using a two-tailed Student’s *t*-test.

### 2.4. Bioluminescence Assays

For bioluminescence assays, WT and *LNK* mutants were transformed with the *pCCA1::LUC* reporter using the floral dip method [38]. Seedlings were grown directly on half strength Murashige and Skoog 0.8% agar medium supplemented with 1% sucrose in a 96-well plate. One seed was placed per well and the seedlings were entrained under 16-h light/8-h dark cycles. After ≈7 days, 40 μL of luciferin (20 mM) were added to each well. The plate was transferred to constant light conditions and placed in a microplate luminometer LB-960 (Berthold Technologies, Bad Wildbad, Germany) to measure the bioluminescence emitted by each seedling every hour. After 5–6 days, data analysis was conducted using the Mikrowin 2000 software (version 4.29, Labsis Laborsysteme GmbH, Neunkirchen-Seelscheid, Germany). Period estimates were calculated with Brass 3.0 software and analyzed using FFT-NLLS. These experiments were performed in duplicate with n = 6 for each genotype. The statistical analysis was done using a two-tailed Student’s *t*-test.

### 2.5. Flowering Time Analysis

For flowering time experiments, the plants were grown on soil at 22 °C under standard long days (LD; 16-h light/8-h dark cycles; 80 μmol·m^−2^ s^−1^ of white light), 12:12 days (12-h light/12-h dark cycles; 80 μmol·m^−2^ s^−1^ of white light), or short days (SD; 8-h light/16-h dark cycles; 140 μmol·m^−2^ s^−1^ of white light) depending on the experiment. Flowering time was estimated by counting the number of rosette leaves at the time of bolting. These experiments were performed in triplicate with n = 12 for each genotype. The statistical analysis was done using a two-tailed Student’s *t*-test.

### 2.6. Hypocotyl Length Characterization

For hypocotyl length measurements, seedlings were grown on 0.8% agar under complete darkness, continuous red light (10 μmol·m^−2^ s^−1^), continuous blue light (2 μmol·m^−2^ s^−1^), continuous white light (LL), or under cycles of white light in SD or LD (all white light treatments 1 μmol·m^−2^ s^−1^). The final length of the hypocotyls was measured 4 days after germination. Light effects on hypocotyl elongation under continuous red and blue light were calculated normalizing hypocotyl length under each light regime to the hypocotyl length of the same genotype under constant dark conditions. For the white light experiments, absolute values of hypocotyl elongation are shown. These experiments were performed in quadruplicate with n = 20 for each genotype. The statistical analysis was done using a two-tailed Student’s *t*-test.

### 2.7. Quantitative Real Time-Polymerase Chain Reaction

For time course analysis, 15-day old plants were grown in Murashige and Skoog 0.8% agar medium under 12-h light/12-h dark cycles at 22 °C, and then transferred for 3 days to continuous white light at 22 °C. Samples were collected every 4 h for 1 day, starting 24 h after the transfer to constant conditions. Total RNA was extracted using TRIzol reagent (Invitrogen, Carlsbad, CA, USA). One microgram of RNA was treated with RQ1 RNase-Free DNase (Promega, Madison, WI, USA) and subjected to retro-transcription with Super Script II Reverse Transcriptase (SSII RT) (Thermo Fisher Scientific, Waltham, MA, USA) and oligo-dT according to manufacturer’s instructions. cDNAs were then amplified with FastStart Universal SYBR Green Master (Roche, Basel, Switzerland) using the Mx3000P Real Time PCR System (Agilent Technologies, Santa Clara, CA, USA) cycler. Samples were pooled across the time series before measuring mRNA abundance to estimate the difference in absolute mRNA levels among genotypes, rather than differences resulting from alterations in the timing of expression of each gene evaluated. The *PP2A* (AT1G69960) transcript was used as a housekeeping gene. Quantitative RT-PCR (qRT-PCR) analysis was conducted using the standard curve method as described in the Methods and Applications Guide from Agilent Technologies. Primer sequences and PCR conditions are available on request. Four biological samples were measured for each genotype.

### 2.8. Biomass and Leaf Morphology Analysis

To analyze leaf morphology, plants were grown on soil at 22 °C in LD conditions (16 h of light/8 h of dark; 80 μmol·m^−2^ s^−1^ white light). When plants were approximately 30-day old, the eighth leaf was cut, scanned, and analyzed with ImageJ [37], and the ratio blade/whole leaf length was calculated. These experiments were performed in triplicate, with n = 10 for each genotype. For size and biomass measurements, plants were grown on soil at 22 °C in SD conditions (8 h of light/16 h of dark; 140 μmol·m^−2^ s^−1^ white light). After bolting, rosettes were photographed and their perimeter determined digitally by drawing the smallest circle that covered the whole rosette using ImageJ [37]. Total aerial vegetative biomass (inflorescence stems removed) was quantified after drying the plants for 3 days at 40 °C in paper wraps. These experiments were performed in duplicate, with n = 18 for each genotype. All statistical analysis was done using a two-tailed Student’s *t*-test.

### 2.9. Shade Avoidance Syndrome Assay

Plants were grown on soil at 22 °C in LD conditions (16 h of light/8 h of dark; 80 μmol·m^−2^ s^−1^ white light) with (treatment) or without (control) a 15-min pulse of far red light, given after the lights were turned off (end-of-day far-red treatment). When plants had 12 rosette leaves, the eighth leaf was cut, scanned, and measured using ImageJ [37]. For hypocotyl length measurements under simulated vegetative shade, seeds were sown on plates containing agar 0.8%, stratified and germinated under LD conditions (16 h of light/8 h of dark; 10 μmol·m^−2^ s^−1^ white light) for 2 days, then transferred to white light conditions with a neutral filter (control) or to white light with a green filter that generated a 0.35 red/far red ratio (treatment). The final length of the hypocotyls was measured after 4 days. Light effects on hypocotyl elongation are shown as absolute values. These experiments were performed in triplicate, with n = 10 for each genotype. The statistical analysis was done using a two-tailed Student’s *t*-test.

### 2.10. Phototropic Response Characterization in Adult Plants and Seedlings

To measure the angle between rosette leaves, plants were grown in soil at 22 °C under SD conditions (8 h of light/16 h of dark; 140 μmol·m^−2^ s^−1^ white light). Six-week old plants were photographed from the side. The angle between the two opposite leaves closer to the light source was measured using ImageJ [37]. These experiments were performed in duplicate, with n = 18 for each genotype. For the phototropic reorientation of leaf blades, plants were grown on soil under LD conditions (16 h of light/8 h of dark; 80 μmol·m^−2^ s^−1^ white light). After the first pair of leaves was fully expanded, plants were exposed to a lateral source of white light (50 μmol·m^−2^ s^−1^). A leaf was considered to have reoriented if the leaf blade was perpendicular to the lateral light source. The number of reoriented leaves was counted in 4-week old plants. These experiments were performed in duplicate, with n = 12 for each genotype. For the characterization of phototropic responses in young seedlings, plants were germinated on vertical plates for 3 days in darkness, and then illuminated from one side with 1 μmol·m^−2^ s^−1^ of blue light for 8–10 h. The plates were scanned to measure the bending angle [39]. These experiments were performed in duplicate, with n = 4 for each genotype. All statistical analysis was done using a two-tailed Student’s *t*-test.

## 3. Results

### 3.1. Loss of Function of *LNK3* and *LNK4* Enhances the lnk1;2 Circadian Clock Phenotype

**Figure 1:**
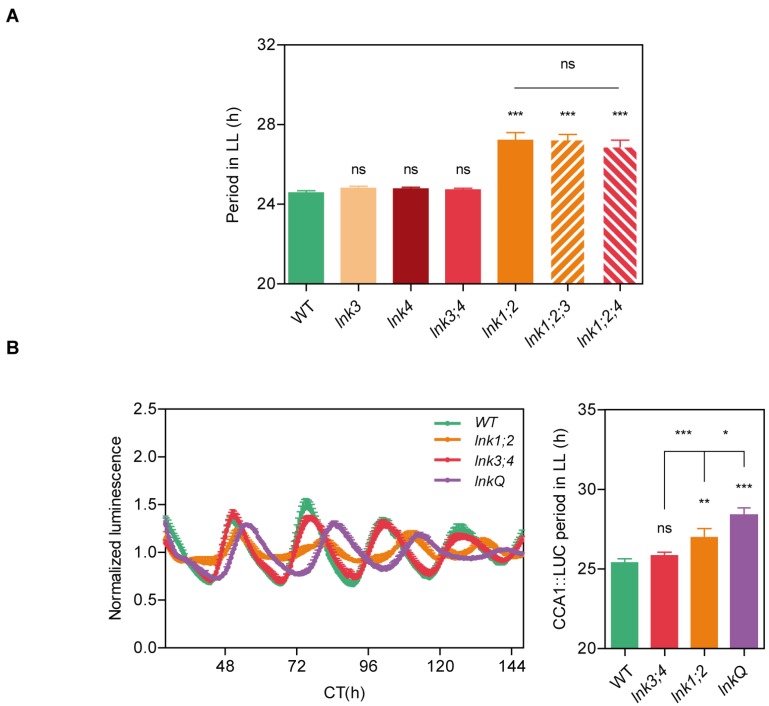
The NIGHT LIGHT–INDUCIBLE AND CLOCK-REGULATED (*LNK*) family exhibits partial redundancy in the control of circadian rhythms. (**a**) Circadian rhythms of leaf movement in continuous light (LL), after entrainment under long-day conditions. (**b**) *CCA1*::LUC activity measured for 5 days in LL, after entrainment under long-day conditions. Bioluminescence was recorded every 2 h over 6 days. Periods of circadian rhythms in leaf movement and *CCA1*::LUC activity were estimated with BRASS 3.0 software (Biological Rhythms Analysis Software System). Error bars indicate standard error of the mean (SEM). Student’s *t*-test was performed between mutants and wild-type (WT) (*: significantly different, *p* ≤ 0.05; **: significantly different, *p* ≤ 0.01; ***: significantly different, *p* ≤ 0.001; ns: not significant).

We previously described several clock- and light-regulated phenotypes for the *lnk1-1;lnk2-1* double mutant. In order to study the degree of redundancy of the LNK family, we identified plants with T-DNA insertions in the *LNK3* (SALK_085551C) and *LNK4* (GK_846C06) loci, and we obtained the triple *lnk1-1;lnk2-1,lnk3-1* (hereinafter referred to as *lnk1;2;3*) and *lnk1-1;lnk2-1,lnk4-1* (hereinafter referred to as *lnk1;2;4*) mutants, as well as the quadruple *lnk1-1;lnk2-1;lnk3-1;lnk4-1* (hereinafter referred to as *lnkQ*) mutant. To assess the functional redundancy among *LNK* genes on the function of the clock, we monitored circadian rhythms of leaf movement in WT plants, *lnk3*, *lnk4*, *lnk3;4*, *lnk1;2*, *lnk1;2;3*, and *lnk1;2;4* mutants using time-lapse photography (Figure 1A). Depletion of LNK3, LNK4, or both did not affect circadian rhythms. As shown previously, *lnk1;2* mutants presented a longer circadian period than the WT (27.2 h vs. 24.5 h, respectively) [29]. Nevertheless, the *lnk1;2* long period phenotype was not affected by depletion of neither *LNK3* nor *LNK4* genes. Interestingly despite showing clear leaf oscillations, an enhanced phototropic response exhibited by the *lnkQ* mutants generated a strong hyponastic phenotype that impaired the quantitative analysis of leaf movements in these plants. In order to surpass this issue, we transformed WT, *lnk1;2*, *lnk3;4*, and *lnkQ* mutants with a bioluminescent reporter and measured oscillations in gene expression driven by the *CCA1* promoter. We found that the circadian period of *CCA1* expression was lengthened in *lnk1;2* (26.9 h) and *lnkQ* (28.4 h) mutants with respect to WT plants (25.4 h). Similar to what we observed for leaf movements, *lnk3;4* double mutants did not display any alterations in the circadian expression of *CCA1* (25.8 h) (Figure 1B). These results indicate that LNK1 and LNK2 played a predominant role in the control of circadian rhythms compared to LNK3 and LNK4, which appeared to play a minor role that was only revealed in the absence of LNK1 and LNK2.

### 3.2. *LNK3* and *LNK4* Depletion in the lnk1;2 Mutant Background Does Not Affect Flowering Time

**Figure 2:**
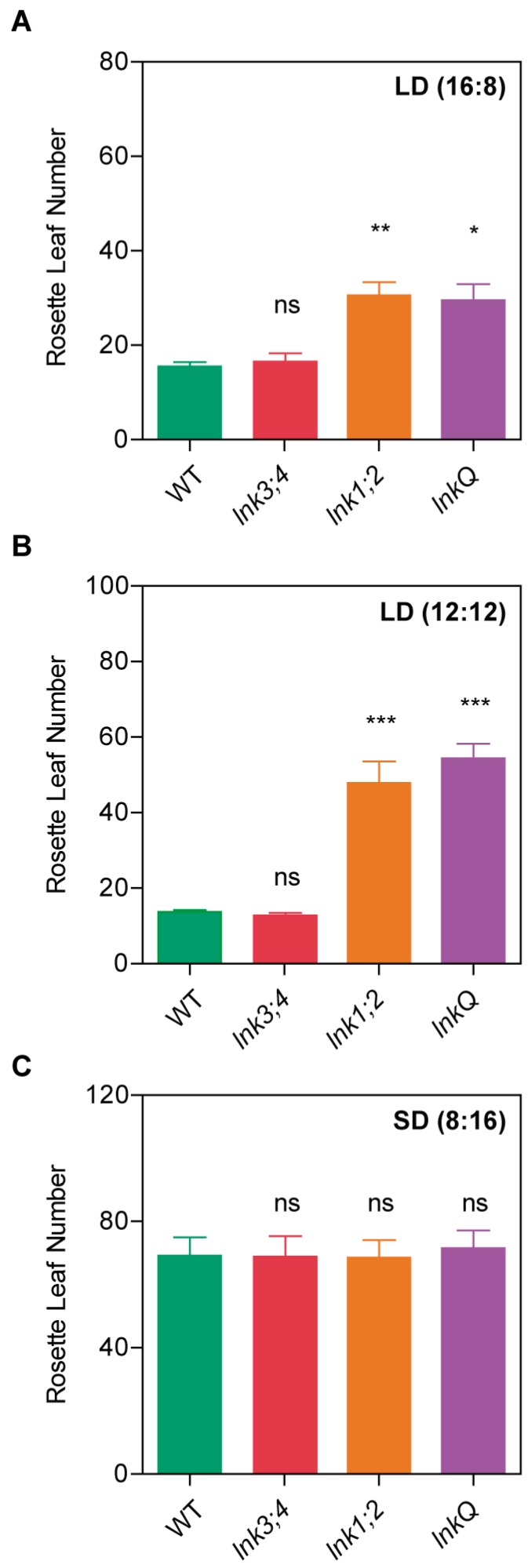
*LNK1* and *LNK2*, but not *LNK3* or *LNK4*, play a role in the photoperiodic control of flowering. Flowering time measured as the number of rosette leaves at bolting in (**a**) long days (LD; 16:8), (**b**) 12 h light: 12 h dark cycles (LD; 12:12), and in (**c**) short days (SD; 8:16). Error bars indicate SEM. Student’s *t*-test was performed between mutants and WT (*: significantly different, *p* ≤ 0.05; **: significantly different, *p* ≤ 0.01; ***: significantly different, *p* ≤ 0.001; ns: not significant).

Another major physiological process that depends on the interaction between the circadian clock and light signaling is the day-length-dependent regulation of flowering time [40]. We already reported that *lnk1;2* double mutants flowered later than WT plants under long-day photoperiods (16 h light/8 h dark). In order to assess the role, if any, of other LNK family members on flowering time regulation, we examined leaf number at bolting in *lnk3*, *lnk4*, *lnk1;2*, *lnk3;4*, *lnk1;2;3*, *lnk1;2;4*, and *lnkQ* mutants. *lnk1;2* and *lnkQ*, but not *lnk3;4* mutants, flowered later than WT plants under either 16:8 h or 12:12 h light:dark photoperiods, whereas no differences in flowering time were observed between *lnk1;2* and *lnkQ* mutants (Figure 2A,B). On the other hand, no delay in flowering time was observed for the *lnk3* and *lnk4* single mutants compared to WT plants, or in *lnk1;2;3* and *lnk1;2,4* triple mutants compared to *lnk1;2* double mutants under a 12:12 photoperiod (Supplementary Figure S1). Finally, none of the mutants analyzed displayed alterations in flowering time under short days (8 h light/16 h dark). These findings suggest that LNK1 and LNK2 were specifically required for the day length-dependent flowering time pathway, rather than for the control of the transition from vegetative to reproductive growth per se, whereas LNK3 and LNK4 play no role in the control of the floral transition.

### 3.3. The *LNK* Family Controls Seedling Photomorphogenesis

**Figure 3:**
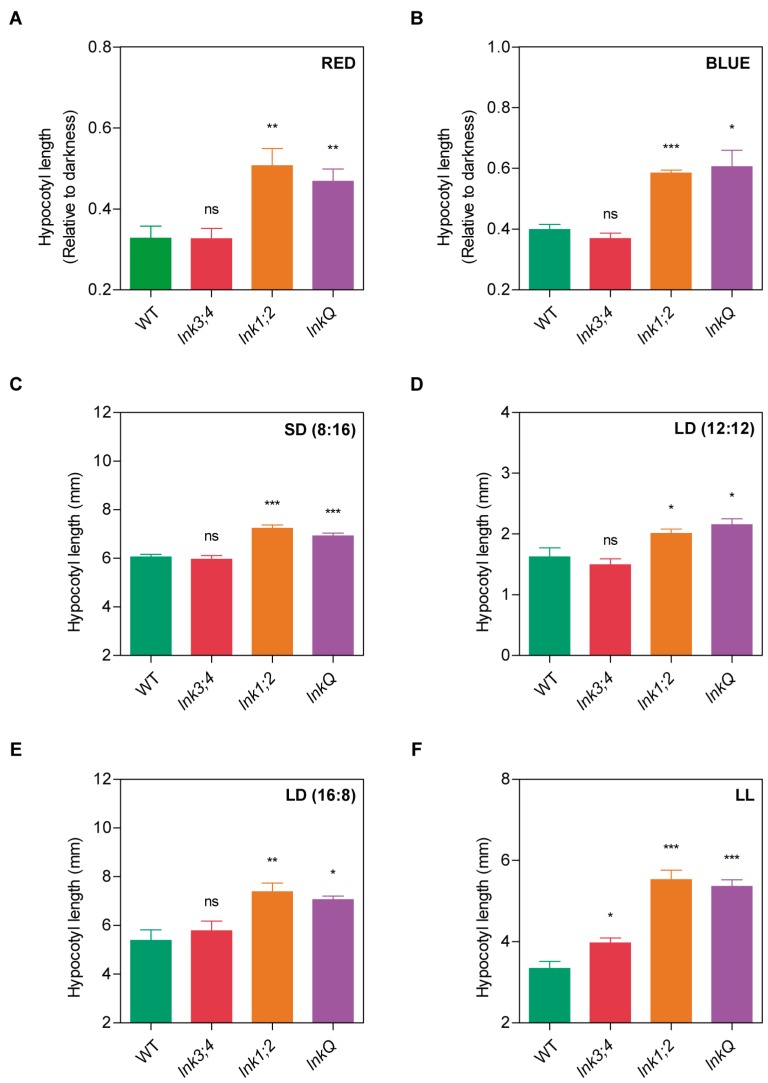
Role of the *LNK* family in the control of photomorphogenesis. Hypocotyls of WT and *LNK* mutants grown under different wavelengths and photoperiods; for continuous red and blue light measurements are expressed relative to the dark control; for white light treatments absolute values are displayed. (**a**) Continuous red light (10 μmol·m^−2^ s^−1^). (**b**) Continuous blue light (2 μmol·m^−2^ s^−1^). (**c**) Short-day photoperiod in white light (1 μmol·m^−2^ s^−1^; SD; 8:16). (**d**) 12 h light:12 h dark cycles in white light (1 μmol·m^−2^ s^−1^; LD; 12:12). (**e**) Long-day photoperiod in white light (1 μmol·m^−2^ s^−1^; LD; 16:8). (**f**) Continuous white light (1 μmol·m^−2^ s^−1^). Error bars indicate SEM. Student’s *t*-test was performed between mutants and WT (*: significantly different, *p* ≤ 0.05; **: significantly different, *p* ≤ 0.01; ***: significantly different, *p* ≤ 0.001; ns: not significant).

Besides flowering time, hypocotyl elongation, an early developmental phenomenon, was also regulated by light and the circadian clock. We first analyzed hypocotyl elongation in darkness. *lnk1;2* and *lnkQ* mutants were only slightly shorter than WT seedlings, while the *lnk3;4* double mutants showed no differences (Supplementary Figure S2), suggesting a minor role for LNK1 and LNK2 in the regulation of cell elongation. We then studied the inhibition of hypocotyl elongation under different light treatments. The *lnk1;2* and *lnkQ* mutants displayed longer hypocotyls than WT seedlings under continuous blue and red light, indicating hyposensitivity to both wavelengths. Nevertheless, no differences were observed between the double and quadruple mutants, nor between the *lnk3;4* double mutants and WT plants (Figure 3A,B). We then assessed the effect of different photoperiods on hypocotyl elongation. Interestingly, both *lnk1;2* and *lnkQ* mutants exhibited longer hypocotyls than WT or *lnk3;4* seedlings under all the photoperiodic conditions evaluated. No significant differences were detected between *lnk1;2* and *lnkQ*, nor between *lnk3;4* and WT seedlings (Figure 3C–E). Finally, we evaluated light inhibition of hypocotyl elongation under continuous white light. Under this condition, all *lnk* mutants displayed significantly longer hypocotyls than WT plants, with *lnk1;2* and *lnkQ* showing longer hypocotyls than *lnk3;4* mutants (Figure 3F). Collectively, these results indicate that LNK1 and LNK2 have a predominant role in the mediation of light inhibition of hypocotyl growth, while LNK3 and LNK4 appear to have a minor, if any, role in this process. This idea is further supported by the fact that *lnk1;2;3* and *lnk1;2;4* triple mutants displayed similar inhibition of hypocotyl growth compared to *lnk1;2* and *lnkQ* under several photoperiods, and by the observation that *lnk3* and *lnk4* simple mutants did not exhibit any alteration at all in hypocotyl elongation (Supplementary Figure S3).

### 3.4. Cumulative Effects of LNK Depletion on the Expression of Core Circadian Clock Genes

**Figure 4:**
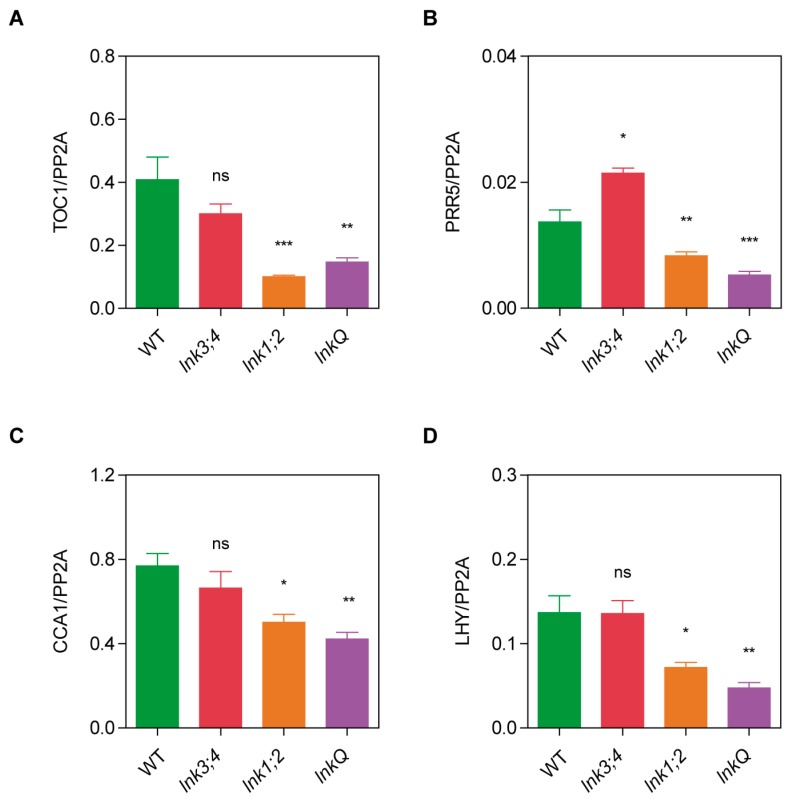
*LNK* family members regulate the expression of central morning and evening clock genes. Mean expression levels were measured using quantitative PCR (qPCR) for the evening genes (**a**) *TOC1* and (**b**) *PRR5*, as well as for the morning genes (**c**) *CCA1* and (**d**) *LHY*. Four biological replicates were used for measuring mRNA abundance. The analysis was conducted after pooling samples collected every 4-h across a time-course series, in order to evaluate difference in absolute mRNA levels among genotypes, rather than differences resulting from alterations in the timing of gene expression. Error bars indicate SEM. Student’s *t*-test was performed between mutants and WT (*: significantly different, *p* ≤ 0.05; **: significantly different, *p* ≤ 0.01; ***: significantly different, *p* ≤ 0.001; ns: not significant).

Although depletion of *LNK3* and *LNK4* lengthened circadian period in the *lnk1;2* mutant background, other light- and clock-regulated processes, such as flowering time and hypocotyl elongation, were not further affected in *lnkQ* compared to *lnk1;2* mutants. This finding revealed a significant degree of redundancy for the *LNK* family in the function of the circadian clock, but a seemingly prevailing role for *LNK1* and *LNK2* in the control of light signaling pathways. To obtain a more detailed understanding of the role of the *LNK* family in the control of the circadian clock, we first analyzed the expression patterns of *LNK3* and *LNK4* genes under free running conditions. Similarly to what was reported for *LNK1* and *LNK2*, *LNK3* and *LNK4* were also rhythmically expressed, with mRNAs showing peak levels in the subjective morning (Supplementary Figure S4) [29]. It is known that LNK1 and LNK2 promote the expression of *PRR5* and *TOC1*, and ultimately these PRRs contribute to the downregulation of *CCA1* and *LHY* at midnight [29,30]. We therefore measured the expression levels of four of the core clock genes in WT, *lnk1;2*, *lnk3;4*, and *lnkQ* mutants. Expression levels of *TOC1* and *PRR5* were reduced in *lnk1;2* and *lnkQ* mutants compared to WT plants (Figure 4A,B). The expression of the morning genes *CCA1* and *LHY* was also reduced in both *lnk1;2* and *lnkQ* mutants, but unaffected in *lnk3;4* mutants (Figure 4C,D). Interestingly, *PRR5* and *CCA1* expression was more severely inhibited in *lnkQ* than in *lnk1;2* mutants, thus revealing some degree of redundancy of the LNKs in the control of gene expression for a sub-set of core clock components.

### 3.5. The *LNK* Family Acts as a Growth Modulator in the Vegetative Stage

**Figure 5:**
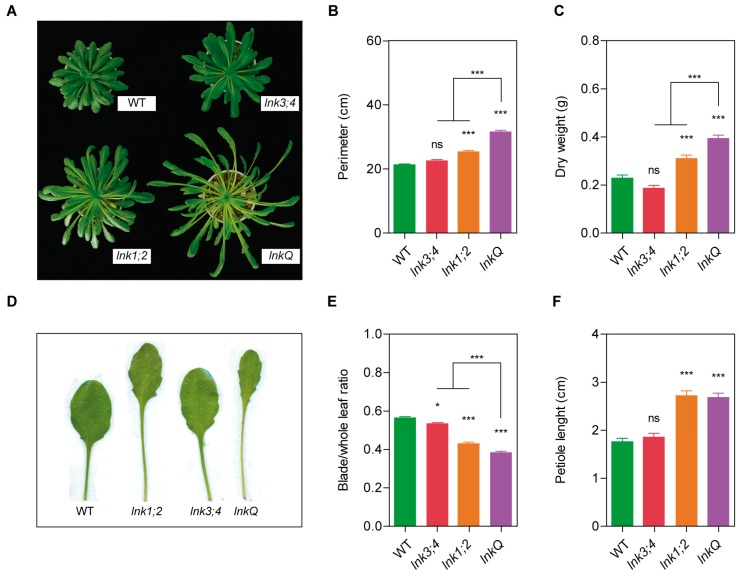
LNK family members act as growth regulators. To assess the role of the LNK family in the control of growth in the vegetative stage, morphological traits of plants grown in short-day conditions were measured. (**a**) Rosette phenotype of 10-week old WT and *lnk* mutant plants grown in a short-day photoperiod. (**b**) Rosette perimeter measured after bolting. (**c**) Dry weight of 10-week old flowered adult plants, inflorescence stems removed. For leaf morphology characterization, plants were grown under long days, and at the 12th-leaf stage, the 8th leaf was cut and measured. (**d**) Representative 8th leaves of WT and *lnk* mutant plants. (**e**) Blade to whole leaf length ratio. (**f**) Petiole length in centimeters. Error bars indicate SEM. Student’s *t*-test was performed between mutant and WT plants (*: significantly different, *p* ≤ 0.05; **: significantly different, *p* ≤ 0.01; ***: significantly different, *p* ≤ 0.001; ns: not significant).

In contrast to the apparent similarity for both flowering time and hypocotyl elongation between *lnk1;2* and *lnkQ* mutants, these plants displayed significant differences in their adult phenotypes (Figure 5A). To further investigate these phenotypic differences, we measured rosette perimeter in plants grown under short-day conditions. The rosettes of *lnkQ* mutants were larger than those of WT plants (31.5 cm vs. 21.3 cm respectively) and *lnk1;2* mutants (31.5 cm vs. 25.3 cm respectively); those of *lnk3;4* double mutants, on the other hand, were similar to WT plants (22.5 cm vs. 21.3 cm respectively) (Figure 5B). We next evaluated biomass accumulation, measured as the dry weight of adult plants grown under short-day conditions. We found that the larger rosettes of *lnkQ* plants were associated with a 72% increase in dry weight compared to WT plants and a 27% increase compared to *lnk1;2* mutants; the *lnk1;2* double mutants exhibited a 36% increase compared to WT plants, whereas *lnk3;4* showed no changes (Figure 5C). Another trait affected in adult *lnkQ* mutants was leaf morphology (Figure 5D). To quantify this, we calculated the leaf blade/total leaf length ratio and measured petiole length of the eighth leaf in plants grown under long-day conditions. All mutants assessed displayed a smaller ratio compared to that of WT plants, indicating smaller leaf lamina and/or longer petioles (Figure 5E). These differences could be attributed to either a difference in leaf blade length, petiole length, or both. Petiole length was similar in *lnkQ* and *lnk1;2* mutants (2.7 cm on average in both mutants) and longer than those of WT (1.7 cm), indicating that the smaller leaf blade/total leaf length ratio observed in *lnkQ* under long-day conditions was due to shorter leaf blades. On the other hand, *lnk3;4* double mutants showed no differences in petiole length compared to WT (Figure 5F). Taken together, these results indicate that the LNK co-activator family redundantly controls petiole length and leaf growth, which in turn impacts rosette size and biomass accumulation in adult plants.

### 3.6. *lnkQ* Mutants Show Normal Shade Avoidance but Enhanced Phototropic Responses at the Adult Stage

**Figure 6:**
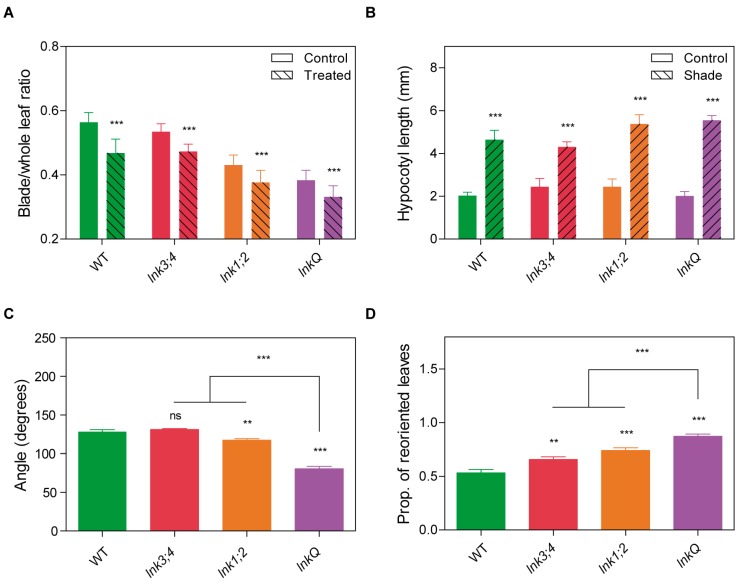
*lnkQ* mutants displayed normal shade avoidance but enhanced phototropic responses. Shade avoidance responses in WT plants and in *lnk* mutants. (**a**) Blade to whole leaf length ratio of the eighth leaf was determined in plants grown in long days and treated or not (control) with a far-red pulse of fifteen minutes after lights went off (end-of-day far-red treatment). (**b**) Hypocotyl length of seedlings grown in white light or white light with an end-of-day far-red treatment. (**c**) For the analysis of leaf angles in adult plants, six-week old plants grown in a short-day photoperiod were photographed from the side. The angle between two opposing leaves closest to the light source was measured. (**d**) The proportion of leaf blades perpendicular to the light source was determined in plants grown in long days with vertical illumination until the first pair of leaves appeared, and then exposed to a lateral light source until the plants developed 8–10 leaves. Error bars indicate SEM. Student’s *t*-test was performed between mutants and WT (*: significantly different, *p* ≤ 0.05; **: significantly different, *p* ≤ 0.01; ***: significantly different, *p* ≤ 0.001; ns: not significant).

The long petioles and small leaf blades of fully grown *lnk1;2* and *lnkQ* mutants resemble those of plants displaying the shade avoidance syndrome [41]. To assess this trait, we exposed WT and mutant plants to an end-of-day far red (FR) treatment, which simulated shade conditions. All genotypes were responsive to simulated shade, showing a smaller leaf blade/whole leaf length ratio than plants grown under control light conditions (Figure 6A). Furthermore, the magnitude of the response was the same in all genotypes (Supplementary Figure S5A). In order to exclude effects of the life stage and the nature of the treatment used for shade simulation, we evaluated the effect of a continuous simulated shade condition under a long-day photoperiod on hypocotyl elongation. Once again, all the genotypes evaluated displayed identical responses to shade simulation, with longer hypocotyls under shade conditions compared to those of control seedlings (Figure 6B and Supplementary Figure S5B). These data suggest that the phenotype of *lnk1;2* and *lnkQ* mutants was not due to constitutive or enhanced responses to shade. Indeed, these findings are consistent with the fact that both mutants, grown under short-day photoperiods, displayed increased biomass accumulation, opposite to the biomass reduction triggered in response to shade signals [41].

Phototropism, perhaps the most recognizable adaptive growth response in plants, is the result of differential cell elongation, which results in the orientation of organs towards the light source to optimize photosynthetic light capture. In flowering plants, phototropism is induced via UV and blue wavelengths (290–500 nm), and modulated to some extent by red light. Remarkably, this phototropic response is evoked over a wide range of light intensities, ranging from extremely small amounts of light to the blue light intensity present on a sunny day under field conditions [42]. Whereas WT plants and *lnk3;4* mutants presented flat rosettes, with angles between opposite leaves of 128 and 131 degrees respectively, *lnk1;2* mutants displayed more erect leaves with an angle of 117 degrees. *lnkQ* leaves formed a notoriously acute angle of 80 degrees, indicating a strong hyponastic phenotype in this high order *lnk* mutant (Figure 6C). Considering the fact that the mutants did not display constitutive or enhanced shade avoidance responses, we hypothesized that the phenotype could be due to an altered phototropic response. We thus scored the reorientation of rosette leaves in plants grown under vertical illumination until the first pair of leaves appeared, and then exposed only to a lateral light source. When plants had 8 to 10 leaves, we measured the proportion of leaves whose blades had re-oriented towards the light source. In WT plants, about half of the leaf blades (48%) adopted such a position, particularly those closer to the light source. Interestingly, all *lnk* mutants tested displayed a significant increase in the proportion of reoriented leaf blades: *lnk3;4* and *lnk1;2* mutants reoriented 60% and 72% of their leaves, respectively, but 87% of the *lnkQ* leaves adopted this arrangement (Figure 6D). To assess phototropic responses at other developmental stages, we measured hypocotyl bending in response to a lateral blue light source, as proposed in Fankhauser et al. [39]. No differences were detected between WT and all *lnk* mutants using this experimental setup, suggesting that these genes redundantly modulate phototropic responses mainly in the adult stage (Supplementary Figure S6).

## 4. Discussion

Our understanding of the plant circadian clock has significantly changed in the last decade, from a relatively simple gene network running on mostly negative transcriptional feedback loops, to a model with several interlocked regulatory gears, in which both activators and repressors play major roles. The recent characterization of the *RVE* and *LNK* gene families has uncovered a new layer of clock activators. Here we analyzed the whole *LNK* family in *Arabidopsis*, and show that different members have distinctive, but also partially redundant, roles in the regulation of circadian rhythms, growth, and development. Significantly, the depletion of the entire family results in a noticeable circadian-period lengthening and remarkable photomorphogenic phenotypes, thus revealing an important role for this gene family as transducers of light signals to the clock.

One of the most important gene families controlling clock function is that of the single MYB transcription factors CCA1, LHY, and the RVEs, grouped in the REVEILLE/LHY-CCA1-LIKE (RVE/LCL) subfamily, are characterized by the presence of a unique LCL protein domain. Interestingly, almost half of the members of this family belong to the RVE clade. While RVE8 and its homologs RVE4 and RVE6 function in a partially redundant manner to modulate the pace of the clock, RVE3 and RVE5 exhibit minor roles in circadian rhythm regulation. Nevertheless, *rve3;rve4;rve5;rve6;rve8* quintuple mutant plants display significant growth alterations compared to WT plants and *rve4;rve6;rve8* triple mutants. Here we explored the smaller family of *LNK* genes. Flowering plants have three main types of *LNK* genes: *LNK1*, *LNK2*, and *LNK3/LNK4* clades. Homologs of *LNK3/LNK4* are found as single copy genes in several plant species, but as duplicates in all crown Brassicaceae. They are likely the product of the most recent genome duplication at the base of this family, which may account for their redundant functions [29].

In a characterization of *LNK1* and *LNK2* as transcriptional co-activators of the circadian core oscillator, Xie et al. reported the interaction of both LNKs with CCA1, LHY, RVE4, and RVE8. *LNK1* and *LNK2* induce *PRR5* transcription through their interaction with RVE4 and RVE8, which, unlike LNKs, have DNA binding domains [30]. Although the LNK family members lack known functional domains, they share two highly conserved regions called R1 and R2. Mutations in those motifs impair the ability of LNK1 to interact with RVE4 [30]. In addition, it was recently reported that all LNKs interact with MYB3, an R2R3-MYB transcription factor that inhibits the *Arabidopsis* phenylpropanoid biosynthesis through the repression of the cinnamate 4-hydroxylase (*C4H*) gene, a key enzyme involved in lignin and flavonoid biosynthesis [43]. Indeed, it is the C-terminal domain common to all LNKs, where the R1 and R2 sites are found, that mediates the interaction with MYB3, and the simultaneous mutation of both R1 and R2 in LNK1 impaired the repressor activity of MYB3 on a *C4H* reporter [43]. Although LNK3 and LNK4 proved unable to act as co-repressors of MYB3 [43], they have recently been shown to interact with RVE8 [31], and both LNK1 and LNK3 interact with the LCL domain of RVE8 [44]. All evidence considered, it is possible that in the absence of LNK1 and LNK2, RVE4 and/or RVE8 may interact with LNK3 and/or LNK4 to promote the expression of PRR5 and TOC1, which would explain the further period lengthening in the *lnkQ* background.

In contrast to what we have seen regarding the control of circadian rhythms, other clock-regulated biological processes, such as flowering time and the inhibition of hypocotyl elongation, were equally affected in both *lnk1;2* and *lnkQ* mutants, suggesting that both LNK1 and LNK2 have a function that LNK3 and LNK4 lack. LNK3 and LNK4 are smaller than LNK1 and LNK2 (≈30 kDa and ≈60 kDa, respectively). Zhou et al. showed that an extra N-terminal tail (ENT) of ≈300 amino acids, present only in LNK1 and LNK2, is necessary for the co-repression of *C4H* by LNK1 and MYB3 [43]. In fact, when the ENT domain of LNK1 was fused to the N-terminus of LNK3, the ENT-LNK3 chimeric protein behaved like LNK1. The ENT domain may also be responsible for the repressive role of LNK1 on the expression of anthocyanin structural genes, such as *UGT79B1*, counteracting in this way the activating function of RVE8 [31]. LNK1 and LNK2 are therefore more complex proteins, whereas LNK3 and LNK4 may be more circumscribed to protein–protein interactions through the R1 and R2 regions common to all members of the family. These differences could explain the predominant roles of LNK1 and LNK2, compared to LNK3 and LNK4, in the regulation of flowering time and the inhibition of hypocotyl elongation.

It is interesting to notice that, in contrast to the case of *rve4;rve6;rve8* and *rve3;rve4;rve5;rve6;rve8* mutants [36], almost every morphologic trait analyzed here was enhanced in the *lnkQ* mutants compared to *lnk1;2*. Both the deletion of RVEs or LNKs trigger an increase in biomass accumulation in comparison to WT plants; these mutant combinations display, however, completely different phenotypes. It was recently shown that LNKs, but not RVE8, interact with the RNA polymerase II and the transcript elongation FACT (facilitates chromatin transactions) complex to rhythmically co-occupy *PRR5* and *TOC1* loci and regulate their circadian transcription. This interaction seems to be important to modulate the transcript initiation and elongation, and is mediated by the R1 and R2 regions [44]. It is thus plausible that the proper transcription of other genes besides *PRR5* and *TOC1* is regulated by the whole LNK family, accounting for such a pleiotropic phenotype.

We found that adult *lnkQ* quadruple mutants have enhanced phototropic responses. Previous work showed that the expression of both *LNK1* and *LNK2* is induced by active phytochromes [29]. In etiolated seedlings, phytochromes do not detect the light gradient per se but they do fine-tune the magnitude of the phototropic response. Phytochromes, with a predominant function of phytochrome A, enhance phototropism modulating the phototropin signaling pathway at several steps in etiolated seedlings [45,46,47]. More recently, it has been shown that shade-induced low red to far-red (R/FR) ratio perceived by phytochrome B enhances the phototrophic response in green de-etiolated seedlings through the action of *PIF* genes and the auxin biosynthetic *YUCCA* genes [48]. The work by Goyal et al. demonstrates that different light signaling pathways modulate phototropic responses in different developmental stages, highlighting the complex crosstalk between the R/FR sensing phytochromes and the blue-light-sensing phototropins [48]. It is therefore possible that LNKs mediate signaling downstream of phytochromes, acting as co-activators and/or co-repressors of the phototropic responses mediated by PIFs. In fact, it was recently shown that the ELONGATED HYPOCOTYL 5 (HY5) transcription factor, a signaling hub downstream of several photoreceptors and a key mediator of photomorphogenesis, binds specifically to the promoters of all *LNK* genes [49].

New insights into circadian clock regulation are emerging as promising avenues for crop improvement. It has been recently shown that an almost complete deletion of the *LNK2* homolog is responsible for the intriguing delay in circadian period observed in domesticated tomato [50,51]. It has been hypothesized that a longer clock period may have been selected as this crop expanded from its center of origin near the equator to higher latitudes. However, the clock pace in local tomato landraces around the equator is also modified compared to sympatric wild ancestors. Considering that biomass increase is a remarkable feature of *lnk* mutants in *Arabidopsis*, it would be interesting to explore whether the circadian phenotype of cultivated tomatoes may be an unintended result of selection on biomass or related traits, that later facilitated the adjustment to different photoperiods. Whereas the increased biomass in plants exhibiting alterations in the circadian clock function may be the gateway to new agronomic applications, it remains to be seen whether this growth promotion brings collateral consequences in other aspects, as proposed in the classical dilemma to grow or defend [52].

## Supplementary Materials

The following are available online at http://www.mdpi.com/2073-4425/10/1/2/s1, Figure S1: Characterization of LNK3 and LNK4 in the control of flowering, Figure S2: Role of the LNK family in the control of hypocotyl elongation, Figure S3: Characterization of LNK3 and LNK4 in the control of photomorphogenesis, Figure S4: Circadian expression of *LNK3* and *LNK4* genes, Figure S5: LNK mutants respond normally under shade conditions, Figure S6: *lnk* mutant seedlings exhibit normal phototropic responses.
