# Supplementary figures and images for: The LNK Gene Family: At the Crossroad between Light Signaling and the Circadian Clock
Source: Genes (Basel). 2018 Dec 20;10(1):2. doi: 10.3390/genes10010002 (PMC6356500; doi:10.3390/genes10010002)

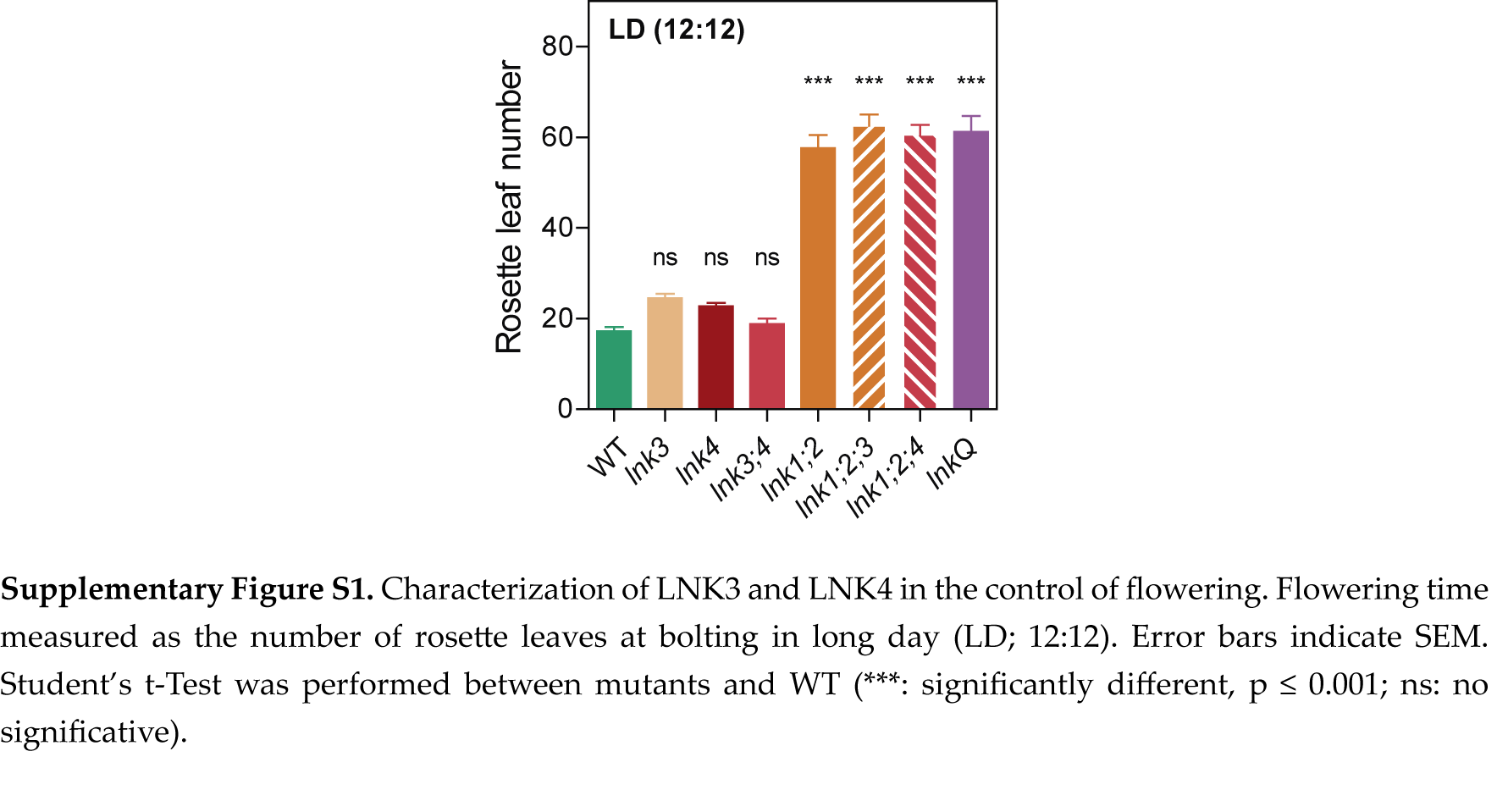

Supplement: Supplementary file 1 [file genes-10-00002-s001.zip › Supplementary_Figures/FIG_S1.tif]

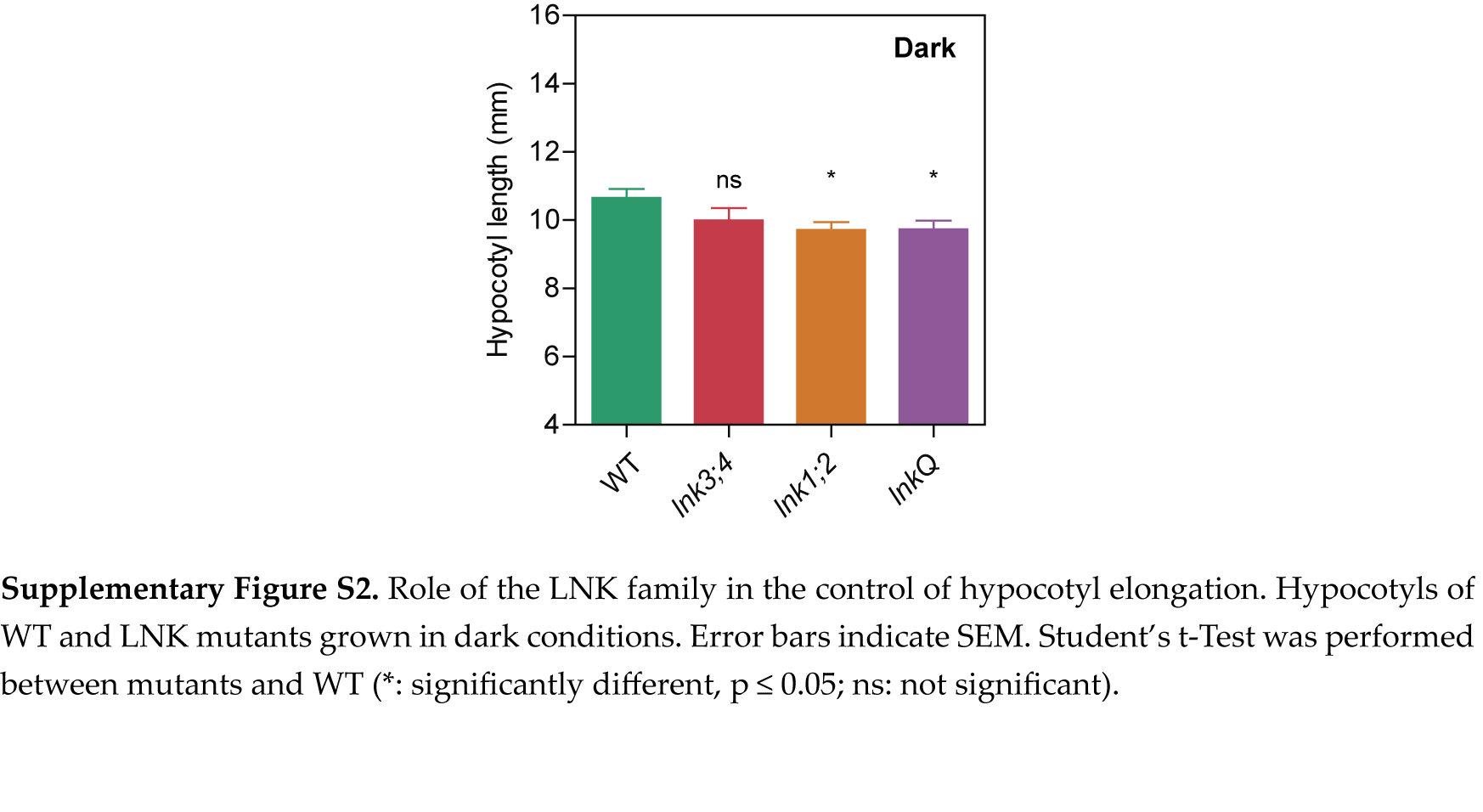

Supplement: Supplementary file 1 [file genes-10-00002-s001.zip › Supplementary_Figures/FIG_S2.tif]

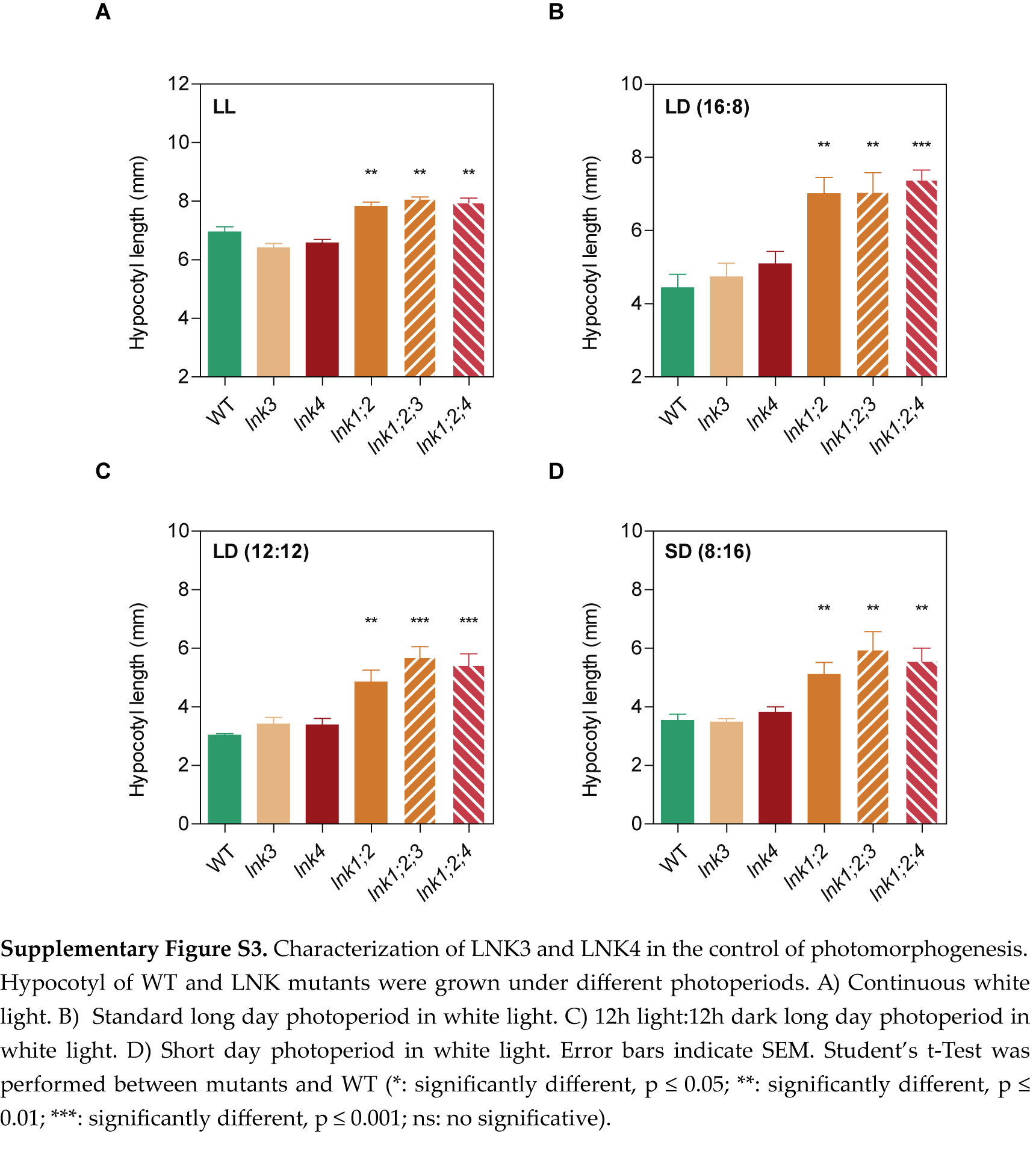

Supplement: Supplementary file 1 [file genes-10-00002-s001.zip › Supplementary_Figures/FIG_S3.tif]

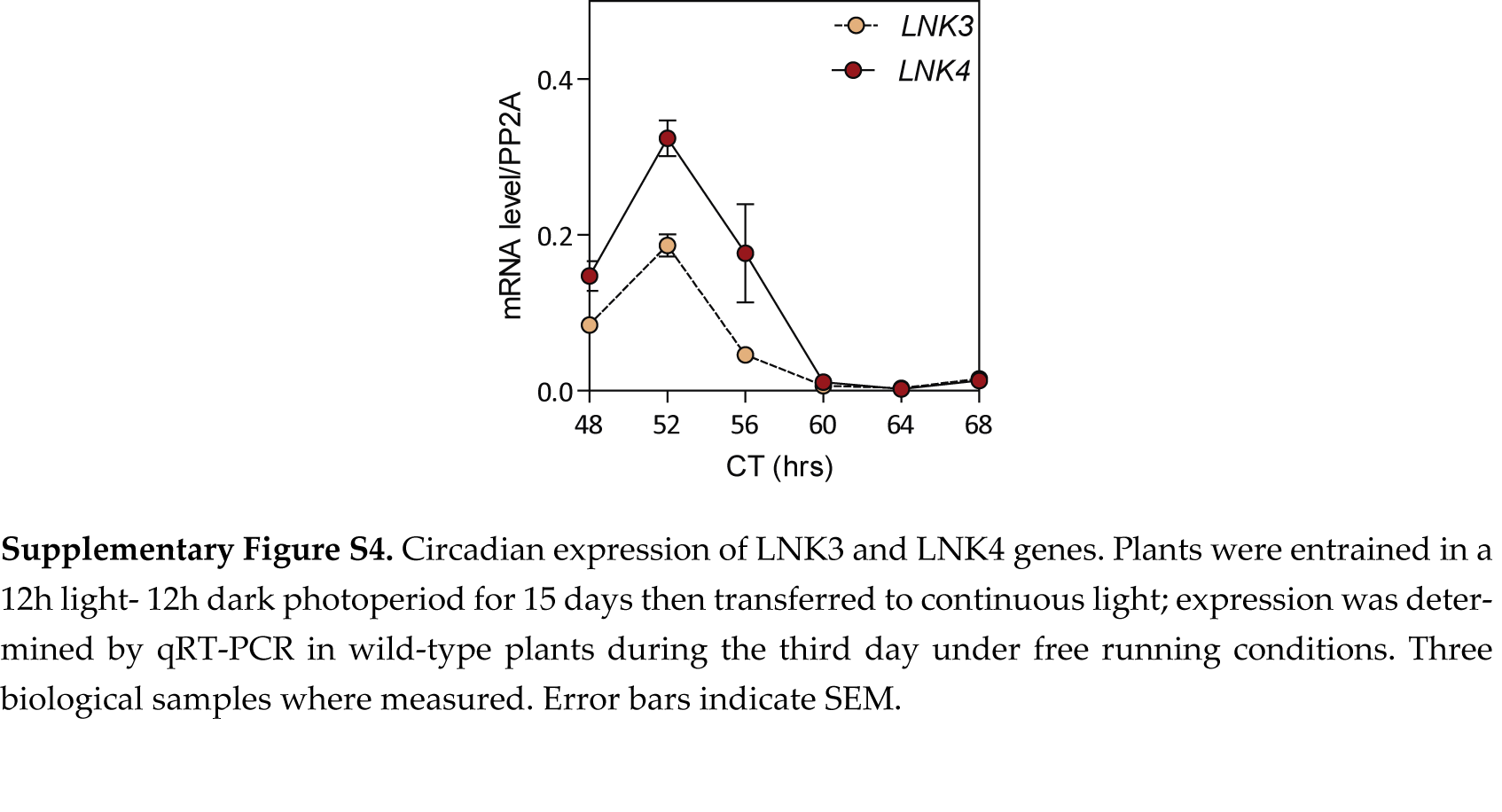

Supplement: Supplementary file 1 [file genes-10-00002-s001.zip › Supplementary_Figures/FIG_S4.tif]

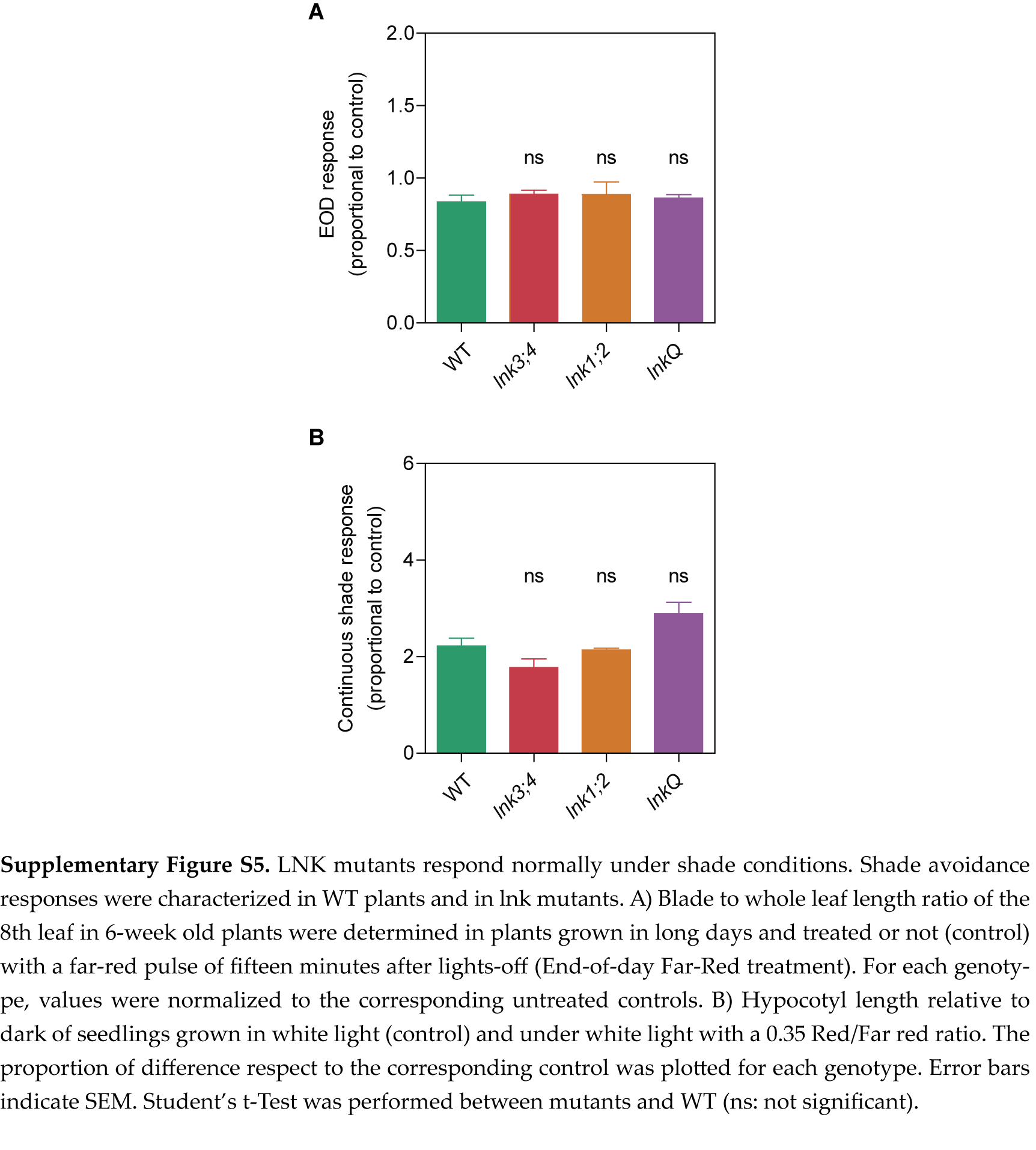

Supplement: Supplementary file 1 [file genes-10-00002-s001.zip › Supplementary_Figures/FIG_S5.tif]

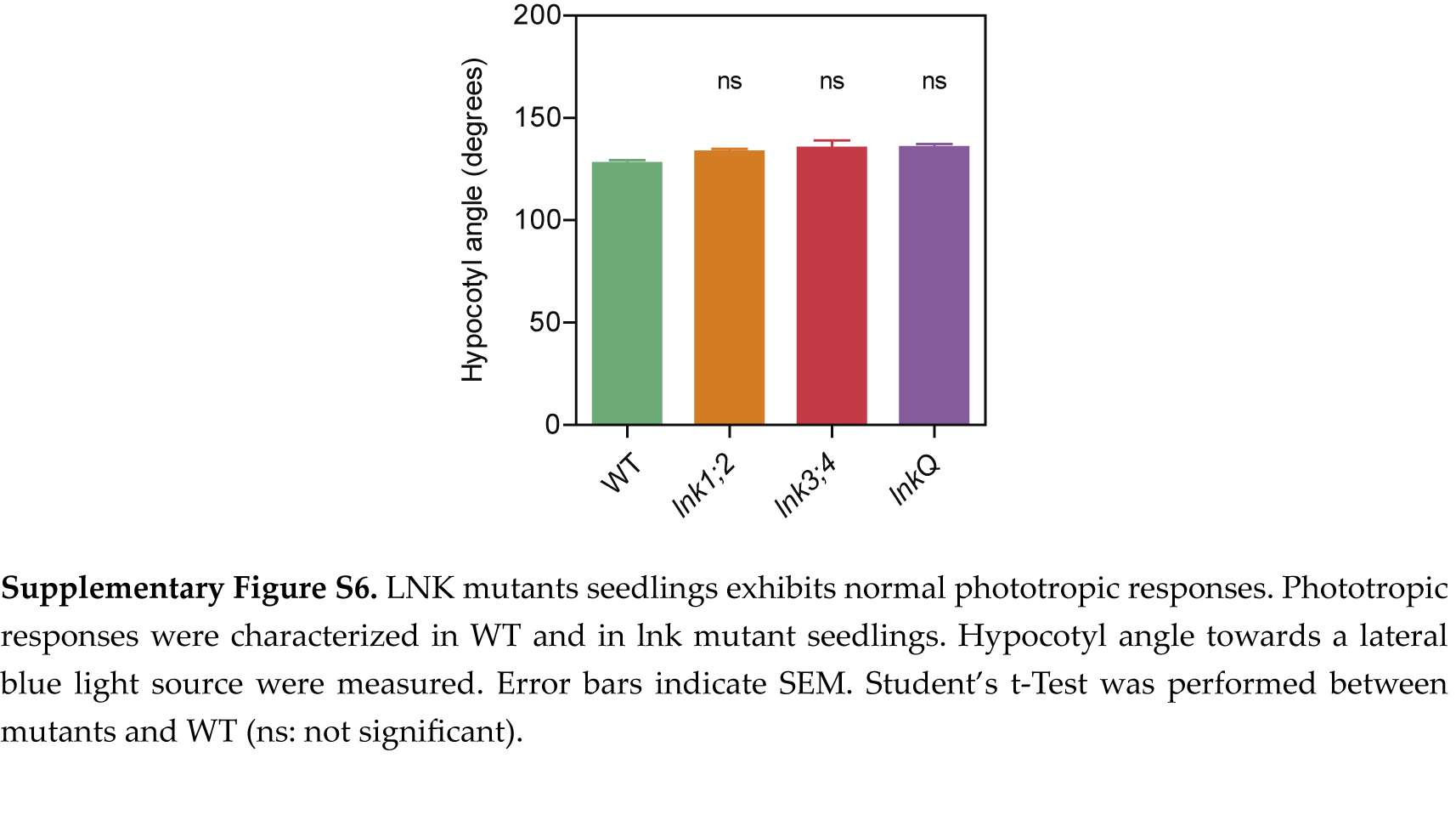

Supplement: Supplementary file 1 [file genes-10-00002-s001.zip › Supplementary_Figures/FIG_S6.tif]
